# Role of GATA3 in Early-Stage Urothelial Bladder Carcinoma Local Recurrence

**DOI:** 10.7759/cureus.44998

**Published:** 2023-09-10

**Authors:** Hristo Popov, Peter Ghenev, George S Stoyanov

**Affiliations:** 1 General and Clinical Pathology, Forensic Medicine and Deontology, Medical University of Varna, Varna, BGR; 2 Pathology, Complex Oncology Center, Shumen, BGR

**Keywords:** early stage carcinoma, urinary bladder, reccurence, gata3, urothelial carcinoma

## Abstract

Background

One of the most characteristic features of non-invasive urothelial carcinoma (UC) is its high recurrence rate. Guanine-adenine-thymine-adenine nucleotide sequence-binding protein 3 (GATA3), as a transcription factor, correlates with urothelial differentiation and has been reported with poor prognosis in high-grade UC and recurrence in breast malignancies. As such, we set out to study the specifics of GATA3 in non-invasive UC, emphasizing on prediction for recurrence.

Methods

The cohort comprised 163 patients, with a follow-up period of five years, including 109 pTa cases and 54 pT1 cases. Immunohistochemical expression of GATA3 was assessed using a histo score (H-score). Kaplan-Meier test was conducted for the time to recurrence, according to the level of expression of GATA3 and the indicators studied. Receiver operating characteristic (ROC) curve analysis was done to determine the role of accuracy and specificity of predictability of the indicators.

Results

Recurrence within the follow-up period was noted in 41.72% of cases. No recurrence relationship was established for age and gender. GATA3 expression showed a varying H-score. Using ROC curve analysis, a cut-off value of 155 divided UC expression levels into low and high, with a sensitivity of 72.7% and specificity of 78.7% (area under the curve=0.800, 95% confidence interval: 0.696-0.904, p<0.001), further showing an association between high levels of nuclear expression and risk of local recurrence (p<0.0001).

Conclusion

Herein we have described the sensitivity of high GATA3 expression in non-invasive UC of the urinary bladder and its relation to local recurrence, independent of gender, age, tumor differentiation, and stage.

## Introduction

One of the most characteristic features in the biological behavior of early-stage urothelial carcinoma (UC) of the urinary bladder is the extremely high recurrence rate compared to malignant epithelial tumors with other localization [1,2]. In its early stages of development - pTa and pT1, patients are subject only to transurethral resection (curettage) and intravesical administration of the Bacillus Calmette-Guérin (BCG) vaccine [1,3,4]. The BCG vaccine heightens the immune response and decreases the recurrence rate, but it remains high. So far, there are no histomorphological or immunophenotypic characteristics of UC that can accurately determine the risk of recurrence [3,4]. Thus, a high-risk group of patients that could potentially benefit from more intensive monitoring with cystoscopy has so far not been defined [1]. The transcription factor guanine-adenine-thymine-adenine nucleotide sequence-binding protein 3 (GATA3) is an immunophenotype marker that is highly specific for UC and is also involved in the recurrence rate of other malignancies, such as mammary, prostate, and pulmonary cancer [5-8]. GATA binding proteins are a family of transcription factors characterized by binding GATA sequences in DNA and regulating cell differentiation and proliferation, including urothelial differentiation as per a 2007 study [9,10]. GATA3 contributes to hematopoiesis, especially to the development and differentiation of T-cells and the morphogenesis of other organs such as the mammary gland and the urogenital system [11-14]. Consistent with the involvement of GATA3 as a key factor in the development of mammary epithelial cells, GATA3 plays a crucial role in carcinogenesis in animal models [14]. GATA3 expression levels have been extensively studied in breast cancer but research in UC especially in regard to recurrence is not sufficient, and the results in the literature are even contradictive [15-17]. Intensive GATA3 expression in high-grade invasive UC has been reported to correlate with a poor prognosis [10]. This gives grounds for a more in-depth study of GATA3 in UC, with emphasis on its role as a prognostic and predictive marker in this location.

With this in mind, we set out to compare the morphological features and GATA3 expression in patients with biopsy-proven UC of the urinary bladder, separated into two groups, with and without a local recurrence with a follow-up period of five years, to establish a histomorphological profile of recurring UC.

## Materials and methods

The study was carried out in a single tertiary healthcare center, St. Marina University Hospital - Varna, Varna, Bulgaria, on in-patient biopsy-proven primary UC of the urinary bladder. All procedures carried out in the study met the ethical standards of the Helsinki Declaration of 1975 and its seventh revision from 2013 and the ethical standards of the Bulgarian Ministry of Healthcare.

Analysis was performed on the primary paraffine-embedded tumor tissue (PETT) used for the definition of the diagnosis, with patient follow-up urinary bladder biopsy used for the definition of recurrence. Patients with a primary diagnosis treated at our center and those diagnosed in our center but monitored at other healthcare institutions were excluded from the analysis.

Study design

A retrospective approach for patient selection was used with the inclusion as mentioned above and exclusion criteria. Primary PETT was reanalyzed for the histological type and degree of differentiation as per the World Health Organization (WHO) Classification of Tumours of the Urinary System and Male Genital Organs, 4th Edition, and tumor stage as per the American Joint Committee on Cancer (AJCC), 8th edition.

The study cohort comprised 163 patients with bladder UC that fit the selection criteria, collected over five calendar years, and separated into two groups, 109 patients in stage pTa and 54 patients in stage pT1.

After the initial biopsy and UC report, all patients received standard protocol treatment with intravesical administration of the BCG vaccine.

After inclusion into the study, both patient groups were prospectively monitored for five calendar years with cystoscopy performed every six months and additional cystoscopy in cases with episodes of hematuria and or dysuria.

Histological slide preparation and assessment

The primary PETT blocks used for the initial diagnosis were cut into four µ thick sections and stained with hematoxylin and eosin (H&E) on a Leica ST5030 Autostainer XL (Leica Biosystems GmbH, Wetzlar, Germany) and with mouse monoclonal IgG anti-GATA3 (HG3-31) antibody (catalog number sc-268, Santa Cruz Biotechnology, Dallas, Texas, US) with a working dilution of 1:250, with immunoperoxidase visualization performed with EnVisiontm FLEX mini kit, high pH (catalog number K8024, Agilent Technologies, Inc., Santa Clara, California, US) on a Dako Autostainer Link 48 (Agilent Technologies, Inc., Santa Clara, California, US) with the preprogrammed staining protocol.

Immunohistochemical expression of GATA3 was assessed using a histo score (H-score) on digital scans of the slides, performed on a Leica Aperio AT2 (Leica Biosystems GmbH, Wetzlar, Germany) with the preprogrammed protocol. H-score was calculated by first defining the intensity (I) of the reaction (0, 1+, 2+, or 3+) and the number of positive cells (P) - 0 (less than 10% positive tumor cells), 1 (10-49% positive tumor cells), 2 (50-74% positive tumor cells), or 3 (more than 75% positive tumor cells). Final H-score calculation was performed using the formula: (1x(% cells with 1+)+2x(% cells with 2+)+3x(% cells with 3+)), with the final result depicted as an absolute value, ranging from 0 to 300. The final H-score was then divided into two groups (low and high) according to the median (determined as the cut-off value).

Statistical analysis

The statistical analysis was performed using the built-in capabilities of Microsoft Excel 2016 (One Microsoft Way, Redmond, Washington, US) and Statistical Package for the Social Sciences (SPSS) statistics version 25 (IBM Corp., Armonk, NY, USA). Descriptive analysis was carried out for mean (μ(X)), standard deviation (SD), minimum (min), and maximum (max) values. Cross-tabulation and chi-square tests were performed for statistically significant differences in the values by category. Statistical significance was considered at p≤0. 05, at 95% confidence. Correlation analysis was performed to analyze the relationship between the values examined and establish their strength. The degree of association was defined as significant at r>0.5<r=0.7, large at 0.7<r=0.9, and extremely large at r>0.9 at statistically significant cut-off values. Kaplan-Meier test was performed to analyze the correlation with the time to recurrence, as per the calculated H-score groups and the clinicopathological indicators. Student's t-test was conducted to compare between indicators and establish any significant differences. Receiver operating characteristic (ROC) curve analysis was carried out to determine the sensitivity and specificity of the studied indicators as predictive factors.

## Results

The mean age of the cases that fit the inclusion criteria (n=163) was 65.92±11.06 years (range 31-89 years), with a male-to-female ratio of 2.71:1 (73%, n=119 males and 27%, n=44 females), and with the highest incidence in the 61-70 age group (38.65%, n=63) (Table 1). No statistically significant difference between the pTa and pT1 groups could be established based on their demographical profile.

**Table 1:** Distribution and characteristics of UC cases UC, urothelial carcinoma

Age group	%	n	Stage (%, n)	Grade (%, n)	Recurrence - stage (%, n)	Recurrence - grade (%, n)
31-40	1.84	3	pTa - 1.23%, n=2	low-grade - 1.84%, n=3	pTa - 0.61%, n=1	low-grade - 0.61%, n=1
			pT1 - 0.61%, n=1	high-grade - none	pT1 - none	high-grade - none
41-50	6.75	11	pTa - 3.68%, n=6	low-grade - 4.29%, n=7	pTa - 2.45%, n=4	low-grade - 2.45%, n=4
			pT1 - 3.06%, n=5	high-grade - 2.45%, n=4	pT1 - 1.23%, n=2	high-grade - 1.23%, n=2
51-60	17.18	28	pTa - 11.04%, n=18	low-grade - 9.82%, n=19	pTa - 4.91%, n=8/	low-grade - 3.68%, n=6
			pT1 - 6.13%, n=10	high-grade - 5.52%, n=9	pT1 - 3.68%, n=6	high-grade - 4.91%, n=8
61-70	38.65	63	pTa - 25.77%, n=42	low-grade - 25.77%, n=42	pTa - 9.20%, n=15	low-grade - 8.59%, n=14
			pT1 - 12.88%, n=21	high-grade - 12.88%, n=21	pT1 - 5.52%, n=9	high-grade - 6.13%, n=10
71-80	28.22	46	pTa - 20.86%, n=34	low-grade - 18.40%, n=30	pTa - 6.75%, n=11	low-grade - 5.52%, n=9
			pT1 - 7.36%, n=12	high-grade - 9.82%, n=16	pT1 - 2.45%, n=4	high-grade - 3.68%, n=6
81-90	7.36	12	pTa - 4.29%, n=7	low-grade - 3.68%, n=6	pTa - 2.45%, n=4	low-grade - 2.45% n=4
			pT1 - 3.06%, n=5	high-grade - 3.68%, n=6	pT1 - 2.45% n=4	high-grade - 2.45% n=4

Histopathology of the cohort showed that 65.6% (n=107) of cases were with low-grade UC and 34.4% (n=56) were with high-grade UC, again with no statistically significant difference between the two groups.

Recurrence within the five-year follow-up period was noted in a total of 41.72% (n=68), with a median age of 47.09±11.31 years (range 35-87 years). A Spearman correlation analysis (rho) was performed, and it again showed no relationship between age (rho=0.053, p=0.504) and gender of patients (rho=0.094, p=0.232) and the occurrence of local recurrence. From the 68 patients in which local recurrence occurred, 63.28% (n=43) were seen in the pTa group and 36.72% (n=25) in the pT1 group, with a statistically significant (p<0.05) predominant occurrence of recurrence in the pT1group. Time to recurrence in the pT1 group was also significantly shorter (p=0.01), with a Kaplan-Meier test establishing 8.12 months progression-free survival (95% CI, 5.16-11.07) compared to the pTa group with a progression-free survival of 11.53 months, (95% CI, 8.03-15.03).

Also, regarding time to local recurrence, the Kaplan-Meier test showed that low-grade UC had progression-free survival (p=0.01), 11.44 months (95% CI, 7.66-15.22), compared to high-grade UC, 8.8 months (95% CI, 5.84-11.75).

**Figure 1:**
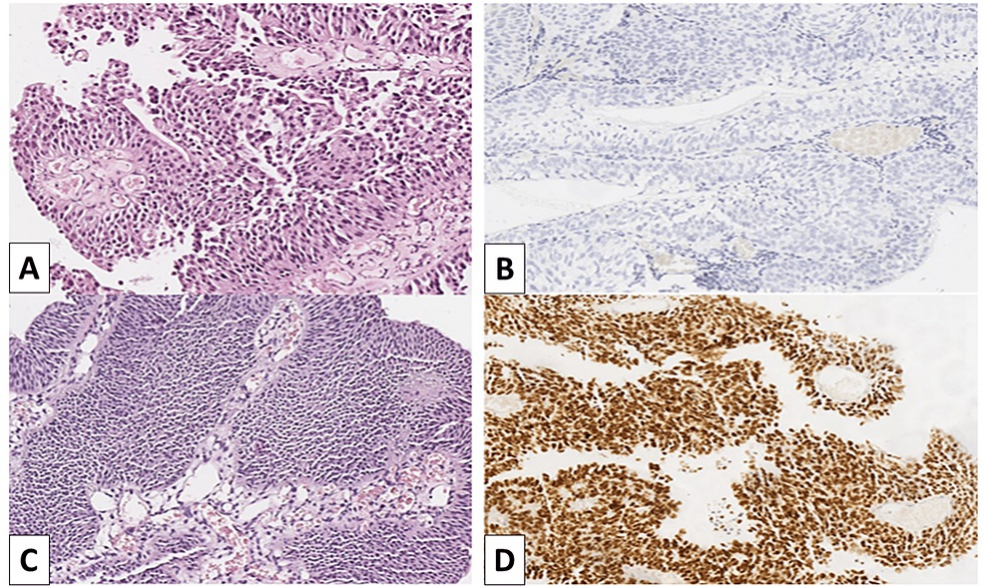
GATA3 expression in recurrent and non-recurrent UCs A. Low-grade non-recurrent UC, H&E staining, original magnification x100; B. No GATA3 expression in low-grade non-recurrent UC, original magnification x100; C. Low-grade recurrent UC, H&E staining, original magnification x100; D. Intensive nuclear expression of GATA3 in low-grade non-recurrent UC, original magnification x100 GATA3, guanine-adenine-thymine-adenine nucleotide sequence-binding protein 3; H&E, hematoxylin and eosin; UC, urothelial carcinoma

**Figure 2:**
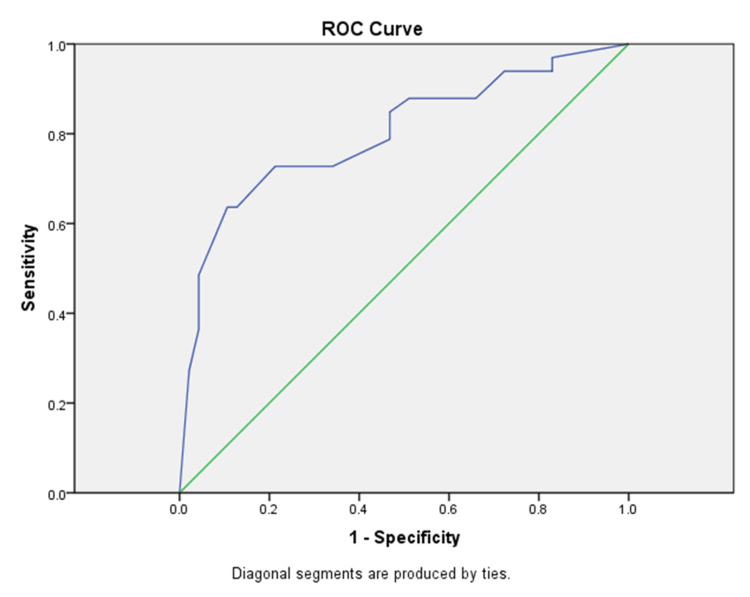
ROC curve analysis and determination of cut-off value for GATA 3 (AUC=0.800, 95% CI: 0.696-0.904, p<0.001) ROC, receiver operating characteristic; AUC, area under the curve; CI, confidence interval

**Figure 3:**
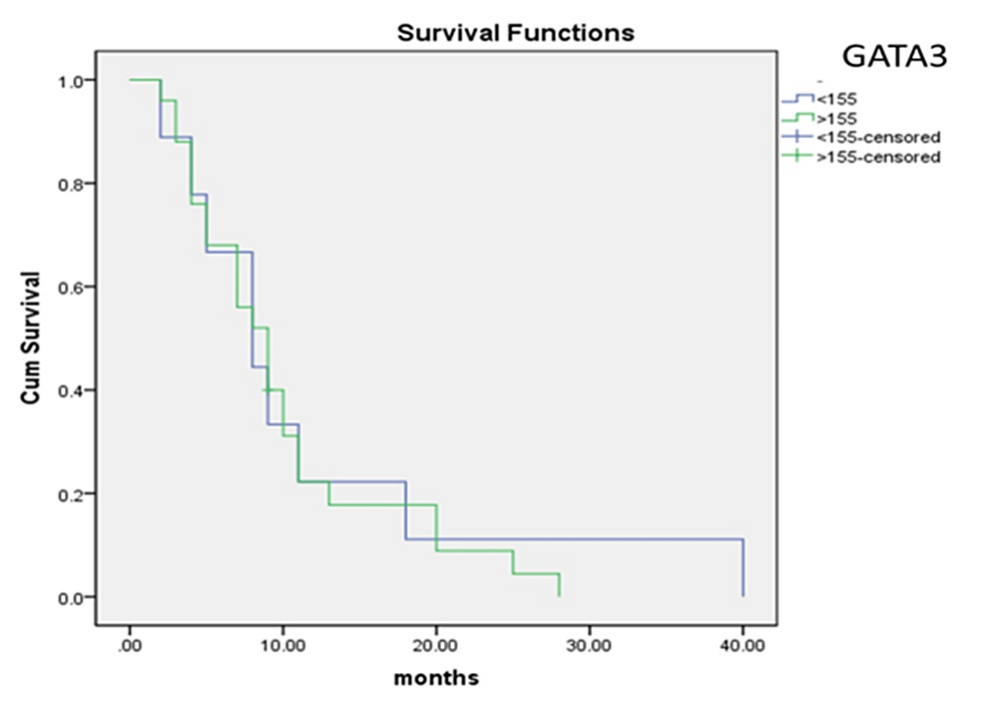
Kaplan-Meier curves of distribution of overall survival of patients with and without recurrence of UC according to GATA3 expression levels GATA3, guanine-adenine-thymine-adenine nucleotide sequence-binding protein 3; UC, urothelial carcinoma

The immunohistochemical study showed a varying H-score of nuclear expression across the cases (Figure 1). Using ROC curve analysis, we determined the H-score cut-off value (155), which divided UC expression levels of GATA3 into low and high, with a sensitivity of 72.7% and specificity of 78.7% (AUC=0.800, 95% CI: 0.696-0.904, p<0.001) (Figure 2). Furthermore, ROC curve analysis showed that there was an association between high levels of nuclear expression of GATA3 (H-score>155) and risk of local recurrence (p<0.0001). The Kaplan-Meier curves show no correlation between H-score and time to local recurrence (p>0.05) (Figure 3).

Further statistical analysis showed no correlation (p>0.05) between the GATA3 H-score and the clinicopathological parameters of UC (gender, age, tumor differentiation, and stage).

## Discussion

Our study included 163 patients with early-stage (pTa and pT1) UC of the urinary bladder, with the highest incidence observed in the age group 61-70 years (n=63), which coincides with the data from Global Cancer Observatory (GLOBOCAN), the WHO, and the National Cancer Registry of Bulgaria, with recurrence within the five-year follow-up period observed in 41.72% (n=68) [18,19]. Тime to local recurrence did not correspond to tumor grade, gender, age, and GATA3 H-score, but there was a statistically significant correlation between high GATA3 expression and the occurrence of recurrence.

GATA3 is a transcription factor that regulates cell differentiation and proliferation, but its role in tumorigenesis has not yet been fully established. Authors such as Gulbinas A et al., 2006, and Engelsen I et al., 2008, found that increased expression of GATA3 in malignancies of the pancreas, endometrial carcinoma, and others negatively impact the degree of differentiation tumor stage and patient prognosis [20,21]. However, some studies link increased GATA3 expression to a more favorable prognosis in tumors such as neuroblastoma [22]. Immunohistochemical expression of GATA3 is widely implemented in practice as a urothelial differentiation marker despite its expression in a myriad of tumors [10,17,23]. In the present study, we established a correlation between the intensity of GATA3 nuclear expression in non-invasive UC of the urinary bladder and local recurrence rates. Despite the statistically significant prediction value of the GATA3 H-score for risk of local recurrence, analysis for time to recurrence did not show any statistical significance. Even so, when comparing the two groups of low-grade and high-grade UC, the time to local recurrence was between 8.8 and 11.44 months, indicating the need for intensive monitoring in high H-score cases at least in the first year. To the best of our knowledge, no study to date has estimated and specified this correlation.

No association was found between GATA3 expression and other clinicopathological parameters such as patient age and gender, degree of tumor differentiation, as well as tumor stage. A probable role of GATA3 expression is also associated with epithelial-mesenchymal transformation, suggesting a higher capacity for UC to invade and produce metastasis [24,25]. This mechanism may also explain the role of GATA3 in local recurrence. According to other authors, Li Y et al. 2014, lower levels of GATA3 expression have been associated with UC with a high grade of differentiation and higher invasive capabilities, which are not reproduced by our findings in a similar setting [25].

As GATA3 also plays a role in the immune response and inflammatory reaction by a myriad of cells is a hallmark of UC, it is crucial to interpret the expression from the UC tumor cells [11,23]. Furthermore, the interactions of GATA3 with other transcription factors and tumor suppressor genes remain unestablished, which is a crucial future direction for this marker with ever-expanding clinical implications [23].

Study limitations

The findings of the present study indicate that there is a higher risk of local recurrence in UCs with high GATA3 expression when compared to those with low expression levels. These findings have several indications for further research into the field and validation. First, as GATA3 is by no means a specific marker for UC and differentiation, a more in-depth look and molecular confirmation should be sought into the biological role of GATA3 proteins in malignant diseases. Second, the expression pattern in recurrent and non-recurrent UCs should be looked at with different clones of poly and monoclonal antibodies, as the lack of expression may be due to the lack of sensitivity of the antibody used by us or the detection of mutant phenotype protein by it. Sadly, in these perspectives, our study falls short due to its initial design. Furthermore, a larger cohort with more extensive follow-up, more advanced stages of the disease, and possible UCs in other locations than the urinary bladder will significantly enrich the medical literature on the topic, far beyond the scope of the present study.

## Conclusions

Herein, we have described the role of increased GATA3 expression by tumor cells from early-stage (non-invasive) UC of the urinary bladder and its relation to local recurrence, independent of gender, age, tumor differentiation, and stage. Even so, GATA3 expression levels do not correlate with time to local recurrence in our study, with an overall follow-up period of five years. Although the suggested mechanisms reveal different aspects of bladder cancer biology and need further clarification, the results presented here provide statistically significant evidence that GATA3 in the primary biopsy is a reliable predictive factor for early relapse.
